# Plantar bullae: an unusual manifestation of bullous pemphigoid

**DOI:** 10.11604/pamj.2025.52.131.49401

**Published:** 2025-11-28

**Authors:** Pawan Banduji Itankar, Gaurav Rajendra Sawarkar

**Affiliations:** 1 Department of Rachana Sharir, Mahatma Gandhi Ayurved College Hospital and Research Centre, Datta Meghe Institute of Higher Education and Research (Deemed to be University), Sawangi (M), Wardha, Maharashtra, India

**Keywords:** Pemphigoid, immunosuppressant, tense bullae

## Image in medicine

A 51-year-old female homemaker from a tropical region presented with a four-month history of tense bullae and erosions on both soles. She initially noticed a few blisters that ruptured, leaving behind painful, raw areas. She used topical antibiotics and sterile dressings, which provided only temporary relief. Over time, the lesions progressed to involve the entire sole, causing significant pain and difficulty with ambulation. She reported no prior history of skin disease or systemic illness. Clinical examination revealed multiple tense bullae of various sizes, some intact and some ruptured, revealing crusted and eroded bases. The bullae were primarily located on the pressure-bearing areas of the sole. Based on the clinical presentation and history, a diagnosis of bullous pemphigoid was considered. Bullous pemphigoid is a chronic autoimmune subepidermal blistering disorder, typically affecting the elderly but can occur in younger individuals exposed to chronic environmental triggers. In tropical agricultural workers, repeated exposure to mechanical friction, ultraviolet radiation, and plant antigens may exacerbate or trigger autoimmune responses. Although bullous pemphigoid usually presents with generalised lesions, localised forms, especially on the lower limbs, can mimic infectious or eczematous dermatoses, potentially delaying diagnosis. Management involves systemic corticosteroids, immunosuppressants (e.g., azathioprine), and topical high-potency steroids. Wound care and infection prevention are critical. This case highlights the importance of considering autoimmune blistering disorders in chronic foot lesions that are unresponsive to conventional treatments, particularly in high-risk tropical populations.

**Figure 1:**
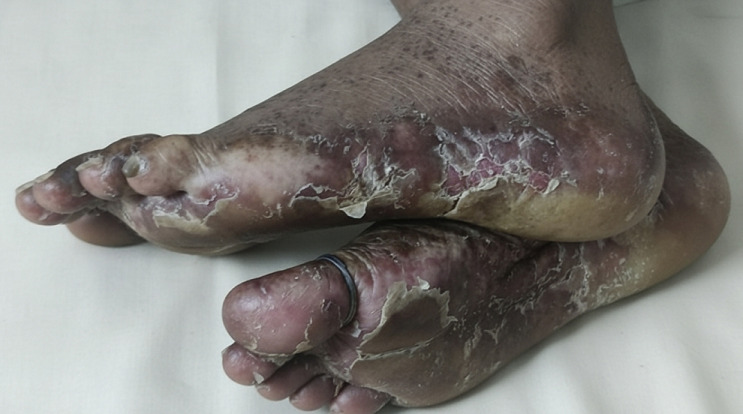
severe, painful, tense bullae and erosions with surrounding erythema on both soles

